# Precision and Customization: The Role of 3D Printing in Modern Prosthodontics

**DOI:** 10.1055/s-0044-1801276

**Published:** 2025-04-16

**Authors:** Haifa Beefathimathul

**Affiliations:** 1 Department of Prosthodontics, College of Dentistry, Qassim University, Buraydah, Qassim, Al-Qassim Region, Saudi Arabia

**Keywords:** prosthodontics, 3D printing, additive manufacturing, dental implants, digital designs

## Abstract

Prosthodontics focuses on the design and fitting of dental prostheses. The advent of three-dimensional (3D) printing has revolutionized this field by transitioning from labor-intensive methods to precise, computer-aided techniques. This review assesses the impact of 3D printing on prosthodontics, highlighting technological advancements, applications, clinical outcomes, and future directions. A literature review was conducted on recent advancements in 3D printing technologies and materials, focusing on their precision and customization capabilities in dental prostheses. 3D printing technologies such as fused deposition modelling, stereolithography, selective laser sintering, continuous liquid interface production, digital light processing, and material jetting offer high precision and customization, enhancing the creation of dental implants, crowns, bridges, removable prosthodontics, orthodontic devices, and maxillofacial prosthetics. 3D printing has improved the accuracy, efficiency, and customization of dental prostheses, leading to better patient outcomes. Multimaterial printing technologies like lithography-based ceramic manufacturing enable the integration of various materials in a single print, further advancing the field. Challenges such as material limitations, cost, and technical expertise remain, necessitating ongoing research and development.

## Introduction

Prosthodontics, a dental specialty, focuses on designing, manufacturing, and fitting artificial replacements for teeth and mouth parts. Prosthodontists restore oral function, comfort, appearance, and health through crowns, bridges, removable prosthodontics, and implants, improving patients' quality of life by addressing tooth loss, congenital defects, or maxillofacial injuries. The American College of Prosthodontists reports that about 120 million Americans are missing at least one tooth, and around 36 million are toothless, indicating a high demand for prosthodontic services. 1 Historically, prosthodontics relied on manual techniques and materials like gold, porcelain, and acrylic resin, involving meticulous craftsmanship and extensive laboratory work. Traditional methods, such as casting and molding for dental crowns and bridges, required multiple patient visits and substantial labor. Gold was preferred for its durability and biocompatibility. However, these manual methods often resulted in inconsistencies in fit and quality. 2

Digital technology has revolutionized prosthodontics, shifting from labor-intensive methods to precise, computer-aided techniques. Computer-Aided Design and Manufacturing (CAD/CAM) has significantly improved the accuracy and efficiency of prosthetic creation. Modern materials like zirconia and lithium disilicate offer superior aesthetics and strength. The digital workflow, from intraoral scanning to milling, allows for quicker turnaround times and enhanced customization. 3

Three-dimensional (3D) printing, or additive manufacturing, creates 3D objects from digital files by layering materials like resin or metal, producing complex shapes and customized designs that traditional methods cannot achieve. 4 Its application in health care, particularly prosthodontics, has grown exponentially. 3D printing allows for highly precise and customized dental prostheses. For instance, Wang et al showed 3D-printed dental implants had superior accuracy and fit compared to conventional implants. 5 Revilla-León and Özcan found that 3D printing reduces production time and material waste, making it cost-effective. 6 Maxillofacial prosthodontics, a sub-specialty within prosthodontics, rehabilitates patients with defects or disabilities affecting the jaw and face, due to congenital conditions, cancer, trauma, or surgery. 3D printing is crucial in this field, enabling the creation of highly customized prostheses that restore function and aesthetics. This technology has revolutionized treatment outcomes by producing patient-specific prostheses tailored to each patient's unique anatomy. 7 The objective of this review is to assess the current state of 3D printing in prosthodontics, examining its technological advancements, applications, clinical outcomes, and future directions. By consolidating findings from recent studies, this review aims to provide a comprehensive understanding of how 3D printing is transforming prosthodontic practices. Furthermore, it seeks to identify the challenges and limitations that must be addressed to fully integrate 3D printing into routine dental care. The scope of the review includes an analysis of various 3D printing technologies, materials used, and their impact on the precision and customization of dental prostheses.

## Advancements in 3D Printing Technology

### Materials Used in 3D Printing

Various materials are utilized in 3D printing for prosthodontics, each offering unique properties that make them suitable for different applications.

#### Acrylonitrile Butadiene Styrene

Acrylonitrile butadiene styrene (ABS) is a thermoplastic polymer known for its strength and durability. Turner et al highlighted that ABS exhibits excellent impact resistance and mechanical properties, making it ideal for dental models and functional prototypes that need to withstand handling and testing. Its robustness and relatively low cost further enhance its suitability for producing dental components requiring high durability. 4 8 This makes ABS a preferred material for applications where both strength and cost-effectiveness are critical. 3 Additionally, ABS' ease of use in various 3D printing technologies, such as fused deposition modelling (FDM), contributes to its widespread adoption in dental prosthetics. 9

#### Polylactic Acid

Polylactic acid (PLA) is a biodegradable thermoplastic derived from renewable resources such as corn starch or sugarcane. Wong and Hernandez noted that PLA is advantageous in dental applications due to its low melting point, ease of printing, and ability to produce fine details. 4 PLA's biodegradability ensures that it is a sustainable option for temporary dental devices and educational models, aligning with eco-friendly practices in modern dentistry. 10 11 Additionally, PLA is compatible with a variety of 3D printing technologies, such as FDM, which further enhances its utility in producing precise and detailed dental models. 8 The material's ability to be composted at the end of its lifecycle makes it a favorable choice for environmentally conscious dental practices. 12

#### Polyethylene Terephthalate Glycol

PETG (polyethylene terephthalate glycol) combines the ease of printing associated with PLA and the strength characteristics of ABS. Tymrak et al emphasized PETG's impact resistance and durability, making it suitable for dental applications requiring robust and long-lasting components. 8 Its chemical resistance and transparency also allow for the production of clear dental models and surgical guides, enhancing the visualization and planning of complex dental procedures. 9 13 PETG's versatility in various 3D printing technologies makes it a valuable material for producing detailed and durable dental prosthetics. 3 4 Additionally, its biocompatibility ensures that it can be safely used in intraoral devices, aligning with the stringent requirements of dental applications. 10

#### Polycarbonate

Polycarbonate is a high-performance thermoplastic with exceptional strength, toughness, and temperature resistance. Hull and Davis demonstrated that polycarbonate's superior mechanical properties make it a reliable choice for dental appliances that need to endure mechanical stress and high temperatures, such as orthodontic devices and surgical instruments. 9 The material's transparency also offers aesthetic benefits, making it a preferred option for clear aligners and other orthodontic applications. 14 Additionally, its biocompatibility and durability ensure long-term performance and safety in dental applications. 15 The versatility of polycarbonate in various 3D printing processes further enhances its utility in producing high-quality, precise dental prosthetics. 16 17

#### Ceramics

Ceramic materials are known for their high strength, biocompatibility, and excellent temperature resistance, making them ideal for permanent dental restorations such as crowns, bridges, and implants. Zhao et al indicated that ceramics used in 3D printing offer superior aesthetic qualities and wear resistance compared to traditional materials. 18 The ability to produce highly detailed and customized dental prostheses with ceramics ensures better integration and longevity, reducing the risk of adverse reactions in the oral environment. 6 19 Additionally, ceramics' high fracture toughness and chemical stability make them suitable for withstanding the harsh conditions of the oral cavity, providing long-term durability and patient satisfaction. 2 7 The use of ceramics in 3D printing also allows for the creation of complex geometries and intricate designs that are difficult to achieve with traditional manufacturing methods, further enhancing their application in modern prosthodontics. 20

#### Polyether Ether Ketone

Polyether ether ketone (PEEK) is a high-performance polymer known for its exceptional mechanical properties and biocompatibility, making it an ideal material for various medical and dental applications. Its strength, stiffness, and resistance to wear and chemicals allow it to be used as a reliable alternative to metals and ceramics in implants and prosthetics. Additionally, PEEK's radiolucency allows for better imaging assessments without interference. Its lightweight nature, along with the ability to customize it through different manufacturing processes like CAD/CAM, further enhances its adaptability in clinical use. 21

### 3D Printing Technologies

Various 3D printing technologies are utilized in prosthodontics, each with unique characteristics and applications.

#### Fused Deposition Modelling

FDM involves melting and extruding thermoplastic filaments layer by layer to build objects. FDM is widely used due to its cost-effectiveness and simplicity; however, it is less precise compared to other methods. This limitation makes FDM more suitable for creating dental models rather than final prosthetics, where higher precision and detail are required. 8 13 Despite its lower precision, FDM's ability to use a variety of thermoplastic materials, such as ABS, PLA, and PETG, makes it versatile for producing durable and functional prototypes. 10 14 Additionally, the ease of use and accessibility of FDM printers have contributed to their popularity in both educational settings and small-scale dental laboratories. 3 22

#### Stereolithography

Stereolithography (SLA) uses a laser to cure liquid resin into hardened plastic in a layer-by-layer process, achieving high precision and smooth surface finishes. This method is particularly ideal for producing dental crowns, bridges, and surgical guides. According to Patel et al, SLA provides better accuracy and surface detail compared to FDM, making it more suitable for intricate dental applications where precision is paramount. 23 Additionally, SLA's ability to produce complex geometries with fine details enhances its utility in creating highly customized dental prostheses. 6 7 The superior surface quality achieved with SLA reduces the need for extensive postprocessing, streamlining the production workflow. 16 Moreover, SLA's compatibility with a wide range of biocompatible resins makes it an excellent choice for producing safe and effective dental appliances. 21 22

#### Selective Laser Sintering

Selective laser sintering (SLS) employs a laser to sinter powdered material, binding it together to form a solid structure. SLS can work with a variety of materials, including metals and ceramics, making it highly versatile. This technique is particularly advantageous for creating durable dental implants and frameworks. As highlighted by Giannatsis and Dedoussis, SLS offers high strength and detail, which are essential for load-bearing dental components. 22 The ability to use metals like titanium in SLS allows for the production of robust and biocompatible implants, providing significant benefits in prosthodontic treatments. 19 24 Additionally, SLS' ability to produce complex geometries and highly detailed structures makes it suitable for customized dental prostheses and surgical guides. 6 25 The technique's compatibility with a wide range of materials further enhances its utility in producing diverse dental applications, from implants to orthodontic devices. 3 22 Table 1 provides a comparative table of FDM,SLA and SLS highlighting their key differences and applications.

#### Continuous Liquid Interface Production

Continuous liquid interface production (CLIP) is a revolutionary 3D printing technology developed by Carbon that enables significantly faster production speeds compared to traditional methods. CLIP utilizes a photochemical process to create solid objects from liquid resin, balancing the interaction of light and oxygen to achieve rapid production. 22 Unlike traditional layer-by-layer methods, CLIP projects ultraviolet (UV) images through an oxygen-permeable window into the resin, creating a “dead zone” of uncured resin that allows for continuous printing without layer steps. This results in superior mechanical properties and smoother surfaces. 12 26 CLIP's efficiency in reducing printing times from hours to minutes makes it ideal for dental models, surgical guides, and other prosthodontic devices. 6 20 The minimized need for postprocessing further enhances its utility in producing high-resolution, detailed parts. 21 24

#### Digital Light Processing

Digital light processing (DLP) is a 3D printing technique renowned for producing high-resolution and detailed prints. DLP uses a digital micromirror device to project light patterns onto a vat of photopolymer resin, curing it layer by layer into a solid object. Each layer is exposed to a precise light pattern, resulting in highly detailed and accurate parts. 26 A key advantage of DLP over other 3D printing technologies is its exceptional resolution and speed. Since the entire layer is exposed to light simultaneously, DLP can produce intricate details and smooth surfaces quickly, making it ideal for applications requiring fine details, such as dental crowns, bridges, and intricate orthodontic models. 7 20 DLP is particularly useful in dentistry due to its ability to produce parts with high accuracy and smooth surface finishes, which are crucial for dental restorations. 6 26 Additionally, DLP's compatibility with a wide range of biocompatible resins enhances its utility in creating customized dental prosthetics and appliances. 16 21

#### Material Jetting

Material jetting (MJT) is a versatile 3D printing technology that excels in producing multimaterial and highly detailed objects. In MJT, print heads deposit droplets of photopolymer materials onto a build platform layer by layer, which are cured by UV light to form solid objects. 12 This technique is similar to inkjet printing but allows for using multiple materials simultaneously. A key benefit of MJT is its ability to print with multiple materials within a single build, enabling the creation of complex parts with varying properties, such as different colors, transparency, and mechanical characteristics. For example, dental models can combine rigid materials for structural components with flexible materials for gum-like textures in a single process. 6 26

MJT's precision and multimaterial capabilities make it ideal for creating detailed and functional dental prosthetics, including implants, removable prosthodontics, and surgical guides. 20 21 The ability to combine different materials in one build enhances the functionality and aesthetics of dental devices, leading to better patient outcomes. 7 16

## Precision and Customization

3D printing technology achieves high precision through layer-by-layer fabrication, which allows for intricate details and complex geometries. This precision is critical for ensuring the accurate fit of dental prosthetics. A study by Osman et al demonstrated that 3D-printed dental restorations had a significantly better marginal fit compared to conventionally manufactured restorations, leading to improved clinical outcomes. 15 One of the primary advantages of 3D printing in prosthodontics is the ability to create patient-specific designs. Customization ensures that dental prosthetics fit perfectly and meet individual patient needs. Liu et al showed that patient-specific 3D-printed dental implants resulted in higher patient satisfaction and better functional outcomes compared to standardized implants. 27

### Postprocessing

Postprocessing plays a crucial role in the precision and fit of dental restorations fabricated through additive manufacturing. This step ensures that the final restoration adheres to the required dimensions, surface smoothness, and mechanical properties. Postprocessing techniques such as polishing, sintering, and surface treatment directly affect the functional performance of restorations, contributing to the accuracy of fit and patient satisfaction.

## Applications of 3D Printing in Prosthodontics

### Dental Implants

3D printing enables the creation of highly customized dental implants and abutments tailored to each patient's anatomical needs. Using digital imaging and CAD software, implants are designed to fit precisely into the jawbone. The process involves taking a digital scan of the patient's oral cavity, virtually designing the implant, and producing it with a 3D printer. Osman et al found that 3D-printed dental implants significantly improved placement accuracy, requiring fewer adjustments during surgery and resulting in reduced operative time and better patient outcomes. 15 Alharbi et al demonstrated the successful use of 3D-printed surgical guides, enhancing precision and patient satisfaction. 28 Patel et al also noted that 3D-printed surgical guides improved implant placement accuracy and reduced surgical time. 23 Revilla-León and Özcan highlighted that 3D printing in dental implants and surgical guides enhances precision and significantly reduces production time and costs. 6

### Crowns and Bridges

The fabrication of crowns and bridges using 3D printing ensures precision and a perfect fit. An intraoral scanner captures the patient's teeth dimensions, creating a digital impression. This scan is imported into CAD software to design the crown or bridge, which is then 3D printed using biocompatible resin or ceramic materials. Postprocessing involves polishing and finishing to achieve the desired aesthetics and fit.

Revilla-León and Özcan noted that 3D-printed crowns and bridges offer a more accurate fit, superior aesthetics, and reduced production time compared to traditional methods. 6 Additionally, Tanis MC et al highlighted better color matching and surface finish, while Dawood et al emphasized the efficiency and cost-effectiveness of the digital workflow. 16 Patel et al and Giannatsis and Dedoussis further supported the advantages of precision and reduced manual adjustments in 3D-printed prosthetics. 23

### Removable Prosthodontics

3D printing technology enables the creation of personalized removable prosthodontics that fit perfectly within the patient's mouth. The process starts with a digital impression of the patient's oral cavity, followed by designing the denture base and teeth using CAD software. The final step involves printing the denture with materials that mimic the natural appearance and function of teeth and gums.

The primary benefits of 3D-printed removable prosthodontics include improved comfort and functionality. Liu et al found that patients fitted with 3D-printed removable prosthodontics reported higher levels of comfort and satisfaction compared to those with conventionally made prosthodontics. 27 The precise fit reduces sore spots and enhances the stability of the prosthodontics, leading to better chewing efficiency and speech. Additionally, Fokkinga et al highlighted the accuracy and customization offered by 3D printing, which improves patient outcomes. 25 Revilla-León and Özcan also noted that 3D printing significantly reduces production time and material waste, making it a cost-effective solution. 6 Furthermore, Dawood et al emphasized the aesthetic benefits and biocompatibility of materials used in 3D-printed removable prosthodontics, ensuring natural appearance and function. 7

### Orthodontic Devices

The production of orthodontic aligners and retainers using 3D printing involves a precise and efficient process. Initially, an intraoral scan captures the exact dimensions of the patient's teeth, providing a highly accurate digital model. This digital model is then used to design the aligners or retainers, ensuring they fit the patient's teeth perfectly. The digital designs are subsequently sent to a 3D printer, which fabricates the devices using clear, biocompatible resin known for its strength and transparency. Once printed, the aligners or retainers undergo a postprocessing phase where they are polished and trimmed to enhance patient comfort and ensure an optimal fit, resulting in effective and comfortable orthodontic devices.

The primary benefits of using 3D printing for orthodontic devices are speed and accuracy. According to Camardella et al the use of 3D printing in orthodontics has significantly reduced the production time for aligners and retainers, allowing for faster treatment planning and delivery. 29 The accuracy of 3D-printed devices ensures a better fit, leading to more effective and predictable orthodontic outcomes.

### Customized Impression Trays

Customized impression trays created with 3D printing technology ensure accurate and detailed impressions of the patient's oral cavity. These trays can be tailored to fit the patient's unique dental anatomy, improving the accuracy of subsequent dental restorations. By using digital impressions and CAD software, the trays are designed to optimize the capture of fine details. Once printed, these trays provide a precise fit, reducing discomfort and enhancing the accuracy of the final dental prosthesis.

Giannatsis and Dedoussis highlighted the benefits of 3D-printed customized impression trays in capturing intricate details and improving the precision of dental impressions. 20 Revilla-León and Özcan noted that the use of 3D printing in creating impression trays significantly enhances the fit and comfort for patients. 6 Patel et al also emphasized that these trays reduce the need for adjustments, streamlining the workflow and increasing efficiency. 23 Alharbi et al demonstrated that 3D-printed trays offer superior consistency and reliability compared to traditional methods, further enhancing the overall quality of dental restorations. 28 Additionally, Dawood et al pointed out that the customization potential of 3D printing allows for better patient-specific adaptations, improving clinical outcomes. 7

### Occlusal Splints

Occlusal splints, used to treat conditions such as bruxism and temporomandibular joint disorders, can be precisely fabricated using 3D printing. Digital scans of the patient's teeth and jaw are used to design the splint, ensuring an exact fit. This precision reduces the need for adjustments and improves the effectiveness of the treatment. 3D-printed occlusal splints are not only more comfortable for patients but also exhibit better durability and customization options compared to traditionally made splints. 7 20 23 28

### Training Models

3D printing technology has significantly enhanced dental education and training by providing highly detailed and accurate models of dental anatomy and procedures. These models can be used for practice surgeries, simulations, and patient education. The ability to print complex structures, such as dental arches and pathological conditions, allows students and professionals to gain hands-on experience in a controlled environment. This improves their skills and confidence in performing actual dental procedures. 6 7 Additionally, Dawood et al noted that 3D-printed models are invaluable in teaching and presurgical planning, providing a realistic and tactile learning tool. 7 Alharbi et al emphasized the role of 3D printing in creating accurate anatomical models that enhance understanding and precision in dental procedures. 17 Patel et al highlighted the cost-effectiveness and accessibility of 3D-printed models for educational purposes, while Martelli et al discussed their impact on improving procedural outcomes and patient safety. 23 24

### Maxillofacial Prosthodontics

Maxillofacial prosthodontics deals with the rehabilitation of patients with defects or disabilities in the maxillofacial region due to congenital conditions, cancer, trauma, or surgery. 3D printing plays a crucial role in creating highly customized and precise prostheses for these patients. Digital imaging and CAD software are used to design prosthetic devices that fit the unique anatomy of each patient. The ability to produce patient-specific prostheses with 3D printing has revolutionized maxillofacial rehabilitation, improving both functional and aesthetic outcomes.

## Multimaterial 3D Printing (LCM Printer)

Lithography-based ceramic manufacturing (LCM) is an advanced 3D printing technology that integrates multiple materials within a single print, enhancing the functionality and aesthetics of dental prostheses. 20 LCM uses a photopolymerizable ceramic slurry, selectively cured with a digital light projector, repeated layer by layer until the object is formed, followed by debinding and high-temperature sintering for full density and strength. 6 18

LCM's multimaterial capabilities combine ceramics with different properties or integrate ceramic and metal components, benefiting complex prosthodontic devices. 12 26 Dental crowns and bridges can have a high-strength ceramic core and an aesthetically pleasing outer layer. 21 Implants can combine biocompatible ceramic surfaces with strong metal components. 19

LCM improves surgical guides, maxillofacial prosthetics, and customized impression trays, offering varied material properties for precision, customized fit for comfort, and enhanced accuracy in impressions. 6 20 24 The primary advantages of LCM include optimized prostheses for strength, wear resistance, and aesthetics creation of patient-specific prostheses reducing assembly and postprocessing and high precision for intricate applications. 16 17 23

### Different Materials in Single Processing

The ability of 3D printing to use different materials in a single process has significantly advanced prosthodontics. Multimaterial printing allows for prostheses with varied mechanical properties, improving functionality and patient outcomes. For example, combining rigid materials for structural support with flexible materials for gum interfaces enhances fit, comfort, performance, and durability. This technique addresses complex clinical needs effectively, providing customized solutions that enhance both aesthetics and function, streamlining production, and ensuring higher precision in dental treatments.

## Conclusion

3D printing has transformed prosthodontics with unmatched precision and customization in dental prostheses. Advances in materials and technologies, such as multimaterial printing, have improved functionality, aesthetics, and patient outcomes. As technology progresses, further integration into routine dental care will overcome current limitations and drive innovation.

**Table 1:** Comparative analysis of FDM, SLA, and SLS technologies in prosthodontics

Factor	FDM (fused deposition modelling)	SLA (stereolithography)	SLS (selective laser sintering)
**Precision**	Moderate precision, suitable for prototyping and basic models	High precision, excellent for detailed and intricate parts	High precision, good for detailed and durable parts
**Material compatibility**	Thermoplastics (e.g., ABS, PLA, PETG, polycarbonate)	Photopolymer resins	Powdered materials (e.g., nylon, metals, ceramics)
**Cost**	Low to moderate cost, affordable for small-scale production	Higher cost, more expensive due to resin and laser technology	High cost, expensive due to powdered materials and laser technology
**Application suitability**	Ideal for prototyping, dental models, and educational purposes	Suitable for dental crowns, bridges, and surgical guides	Suitable for dental implants, frameworks, and durable components
**Surface finish**	Rougher surface finish, may require postprocessing	Smooth surface finish, minimal postprocessing required	Rougher surface finish, usually requires postprocessing
**Build speed**	Moderate, dependent on model complexity	Faster for small parts, slower for large parts	Moderate, dependent on part density and complexity
**Strength and durability**	Lower strength, suitable for non-load-bearing applications	Good strength, suitable for functional prototypes	High strength, suitable for load-bearing applications
**Ease of use**	User-friendly, widely accessible, and easy to operate	Requires more technical knowledge and handling	Requires significant technical expertise and handling
**Postprocessing**	Often necessary to improve surface quality	Minimal, mainly for cleaning and curing	Required for removing excess powder and surface finishing
